# Binding Mode Exploration of B1 Receptor Antagonists’ by the Use of Molecular Dynamics and Docking Simulation—How Different Target Engagement Can Determine Different Biological Effects

**DOI:** 10.3390/ijms21207677

**Published:** 2020-10-16

**Authors:** Marica Gemei, Carmine Talarico, Laura Brandolini, Candida Manelfi, Lorena Za, Silvia Bovolenta, Chiara Liberati, Luigi Del Vecchio, Roberto Russo, Carmen Cerchia, Marcello Allegretti, Andrea Rosario Beccari

**Affiliations:** 1 Dompé Farmaceutici SpA, via Campo di Pile, 67100 L’Aquila, Italy; carmine.talarico@dompe.com (C.T.); laura.brandolini@dompe.com (L.B.); candida.manelfi@dompe.com (C.M.); marcello.allegretti@dompe.com (M.A.); andrea.beccari@dompe.com (A.R.B.); 2 Axxam, Via Meucci 3, Bresso, 20091 Milano, Italy; lorena.za.lz@axxam.com (L.Z.); silvia.bovolenta.sb@axxam.com (S.B.); chiara.liberati.cl@axxam.com (C.L.); 3 Ceinge Biotecnologie Avanzate, via G. Salvatore 486, 80145 Napoli, Italy; prof.luigi.delvecchio@gmail.com; 4 Department of Pharmacy, University of Naples “Federico II”, via D. Montesano, 49, 80131 Napoli, Italy; roberto.russo@unina.it (R.R.); carmen.cerchia@unina.it (C.C.)

**Keywords:** bradykinin 1, allosteric inhibitors, biased signaling, neuropathic pain

## Abstract

The kinin B1 receptor plays a critical role in the chronic phase of pain and inflammation. The development of B1 antagonists peaked in recent years but almost all promising molecules failed in clinical trials. Little is known about these molecules’ mechanisms of action and additional information will be necessary to exploit the potential of the B1 receptor. With the aim of contributing to the available knowledge of the pharmacology of B1 receptors, we designed and characterized a novel class of allosteric non-peptidic inhibitors with peculiar binding characteristics. Here, we report the binding mode analysis and pharmacological characterization of a new allosteric B1 antagonist, DFL20656. We analyzed the binding of DFL20656 by single point mutagenesis and radioligand binding assays and we further characterized its pharmacology in terms of IC_50_, B1 receptor internalization and in vivo activity in comparison with different known B1 antagonists. We highlighted how different binding modes of DFL20656 and a Merck compound (compound 14) within the same molecular pocket can affect the biological and pharmacological properties of B1 inhibitors. DFL20656, by its peculiar binding mode, involving tight interactions with N114, efficiently induced B1 receptor internalization and evoked a long-lasting effect in an in vivo model of neuropathic pain. The pharmacological characterization of different B1 antagonists highlighted the effects of their binding modes on activity, receptor occupancy and internalization. Our results suggest that part of the failure of most B1 inhibitors could be ascribed to a lack of knowledge about target function and engagement.

## 1. Introduction

Kinins mediate their functions through two subtypes of 7 transmembrane (TM) G-protein-coupled receptors (GPCRs) named kinin receptors B1 and B2. Kinin receptor activation exerts several biological effects, including cell proliferation, leukocyte activation, cell migration, endothelial cell activation and nociception, which have been associated with pain, pathologic inflammatory processes and, more recently, cancer [1]. The B2 receptor appears to be involved in the acute phase (hours) of the inflammatory and pain response and the B1 receptor sustains the chronic phase (days) of signaling [2,3,4,5]. To exert their different roles, B1 and B2 receptors have different expression and regulation patterns. The B2 receptor is constitutively expressed by several cell types and its agonist-induced activation is rapidly followed by desensitization through receptor phosphorylation, β-arrestin recruitment, G-protein uncoupling, receptor internalization and recycling [6]. In contrast, the B1 receptor is expressed at low levels under physiological conditions but it is highly induced by tissue injury, bacterial endotoxin and inflammatory mediators such as cytokines. When B1 expression is induced, its activity is constitutive and its signaling shows very little desensitization [7]. Binding of an agonist to the B1 receptor reduces its constitutive internalization, thus lowering the rate of receptor clearance on the cell membrane and subsequent degradation [6,8,9,10,11]. Because of its role in chronic inflammation and pain, the B1 receptor is an attractive target for analgesic drug development [12,13], as demonstrated by upcoming trials [14,15]. In almost 30 years of studies of the B1 receptor and its antagonists, a large number of inhibitors was described, such as arylsulfonamide- and amide-based small molecules, with some of them reaching phase II clinical trials as Sanofi SSR-240612 and Merck MK-0686 [16,17]. Over the last 10 years, with the evolution of our knowledge of GPCR biology, the relevance of the binding mode and mechanism of action has become clear, together with the concept of functional selectivity and biased ligand binding. In this respect, the design of novel B1 allosteric inhibitors and the deep characterization of the acting mode in comparison with a reference compound could provide new insights into the ideal characteristics of B1-targeting molecules.

With respect to ligand binding, many studies identified in the 7TM receptors a main ligand-binding pocket located between the extracellular segments of TM-III, -IV, -V, -VI and -VII and secondary interaction sites, mainly in the TM-V and -VI minor pocket, which is involved in fine tuning of receptor activation and biased signaling [18]. Our previous studies revealed the presence of this conserved allosteric site in other GPCRs, such as C-X-C Motif Chemokine Receptors 1 and 2 (CXCR1 and CXCR2) and Complement component 5a (C5a) [19,20] and suggested a key role of the binding mode in this pocket in the fine modulation of the receptor internalization process. Because receptor internalization is the key for ligand scavenging, a different molecular mechanism of action could imply different pharmacological in vivo behavior, with a potentially tremendous impact in chronic conditions where progressive ligand accumulation may revert the inhibitory effect. Here, we report the pharmacological characterization of a new allosteric B1 receptor inhibitor, DFL20656, endowed with nanomolar activity, high residence time on the B1 receptor and the ability to induce receptor internalization, opposing agonist activity, by virtue of its binding mode in the minor pocket. Our results demonstrate that DFL20656 is a novel allosteric competitive inhibitor with persistent receptor occupancy and the ability to induce internalization of the B1 receptor. These results further call for the importance of a deep in vitro characterization of the ligand binding properties of GPCR modulators as integrating part of the lead selection process to avoid confounding in vivo results.

## 2. Results

### 2.1. Multiple Receptor Conformations Molecular Docking Experiments

**Figure 1:**
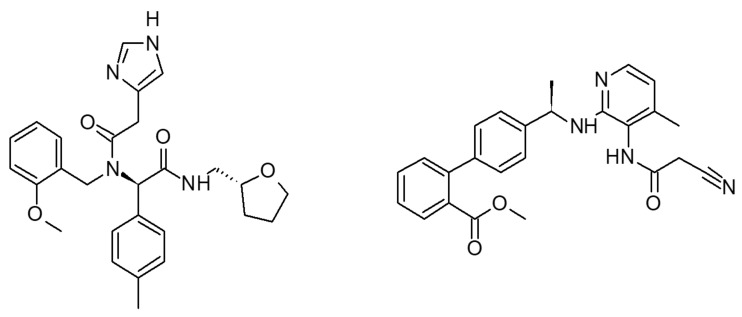
Chemical structures of DFL20656 (on the left) and Merck compound 14 (on the right).

Our receptor/ligand recognition studies were based on a high-quality homology model of the B1 receptor generated with Iterative Threading ASSEmbly Refinement (I-TASSER), refined with the Maestro Macromodel and then energetically minimized by molecular dynamics (MD) simulations of the receptor in the membrane [21]. The MD calculation was evaluated monitoring the Root mean square deviation (RMSD) values, computed on backbone atoms and the total energy of the system (Figure S2, Supplementary Materials). The initial 25 ns were necessary to the complex to equilibrate itself. Then, the RMSD achieved a good structural stability (RMSD values stabilize around 2 Å after 30 ns). After the MD quality assessment, the receptor conformers were extracted from MD trajectory every 10 ns (every 50 frames). DFL20656 and the Merck compound 14 [22], a previously reported B1 receptor antagonist chosen for comparison, (Figure 1) were docked on the B1 homology frames based on a progressive ensemble docking strategy, which involved different B1 conformations.

**Figure 2:**
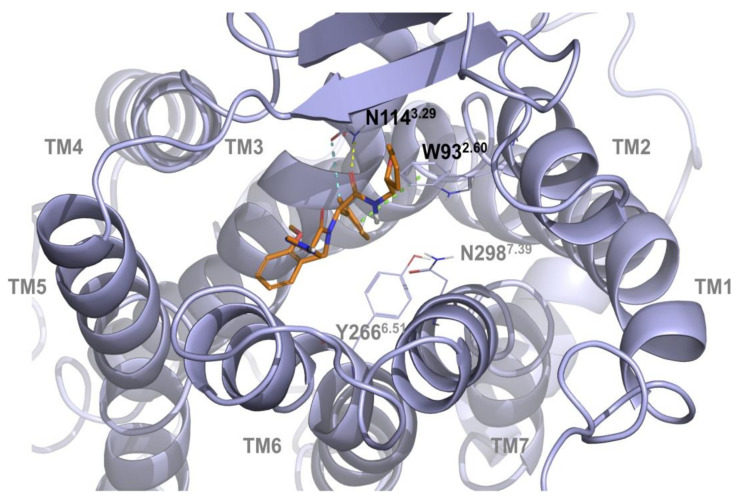
3D representation of DFL20656 binding the B1 receptor pocket. DFL20656 is represented in orange sticks, protein and binding site residues (colored in slate) are represented as cartoon and lines respectively, whereas hydrogen bond, aromatic hydrogen bond and pi-pi interactions are reported in yellow, cyan and green dashes respectively. In the figure are shown the interaction of the carbonyl group of amidic region and methylphenyl group of DFL20656, with N114^3.29^ and W93^2.60^ residues, respectively. Labels are reported with Ballesteros–Weinstein numbering scheme.

Docking results confirmed that the two compounds share the same region of the binding site, highlighting the importance of specific anchor points that were previously reported to be necessary for allosteric inhibition by our group [19] and specifically reported for B1 receptor inhibition in a recent paper [23]. Docking simulation highlighted how the compound DFL20656 interacts with N114^3.29^ and W93^2.60^ (Figure 2).

**Figure 3:**
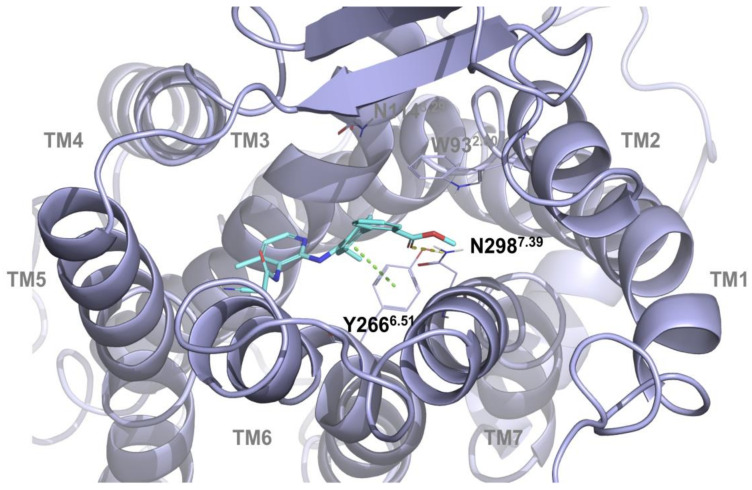
3D representation of Merck compound 14 binding the B1 receptor pocket. Merck compound 14 is reported in cyan sticks, protein and binding site residues (colored in slate) are represented as cartoon and lines respectively, whereas hydrogen bond and pi-pi interactions are reported in yellow and green dashes respectively. In the figure are shown the interaction of the carbonyl moiety of methyl ester group with N298^7.39^ and the pi-pi interaction between the Merck compound and residue Y266^6.51^. Labels are reported with Ballesteros–Weinstein numbering scheme.

In particular, the carbonyl group of acetamide region and methylphenyl group of DFL20656 are strongly anchored to the N114^3.29^ residue via two hydrogen bonds and a pi-pi- stacking interaction with W93^2.60^ as shown in Figure 2. The specificity of this residue taking part in this interaction pattern confers a certain biological behavior to DFL20656 on the B1 receptor. The different binding mode and residue interactions of the Merck compound 14 are shown in Figure 3.

### 2.2. Validation of the Binding Site and Binding Mode through Single Point Mutagenesis and Radioligand Binding Experiments

**Figure 4:**
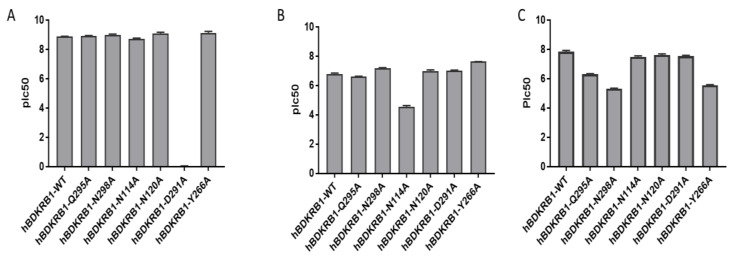
Effect of single point mutations on compound activity. [Leu^8^]-Lys-desArg^9^-BK activity is affected only by D291A, which is known to be a key residue for orthosteric cavity (**A**). DFL20656 and Merck compounds are affected by mutations inside the minor pocket but with a different pattern, to note that both are unaffected by the D291A mutation. DFL20656 presents a single key interaction with the N114A (**B**). The Merck compound 14 is not affected by N114A mutation by engaging the minor pocket with a different pattern of interactions, in fact its activity is reduced by N298A, Y266A and Q295A (**C**). The data represent the mean of six experimental replicates. All data were presented as the mean ± SEM. Bonferroni-Dunn post hoc test was used. *p* < 0.05 was considered representative of significant differences.

We performed single point mutagenesis of the human isoform of the B1 receptor inside the identified allosteric binding site to characterize the binding mode of DLF20656. From our previous knowledge of the minor pocket of GPCR receptors, we selected six amino acids for the single point mutagenesis [19]. Q295A, N298A, N114A, N120A and Y266A were chosen to observe the binding of the molecules in the minor pocket, while D291A was chosen to evaluate binding to the orthosteric site [24]. As shown in Figure 4B, the N114 residue is fundamental for DFL20656 binding, while N298 had a negative effect on binding since its mutation resulted in enhanced activity of the molecules with respect to the wild-type receptor. The Merck compound 14 demonstrated binding to the same allosteric site but with a different interaction pattern, as Q295A, N298A and Y266A strongly affected its activity, while N114A did not (Figure 4C). As expected, [Leu^8^]-Lys-desArg^9^-BK engaged the receptor in the orthosteric site, so only the D291A mutation affected its activity (Figure 4A).

Thus, the binding mode hypothesized by mean of docking simulation (Figure 2; Figure 3) is consistent with the single point mutagenesis results. Through radioligand displacement binding experiments, we revealed that, although our compound DFL20656 binds to an allosteric site, its behaviour was competitive since it was able to completely displace the radiolabelled orthosteric agonist in binding experiments (Table 1 and Figure S1, Supplementary Materials).

From the radioligand binding experiment performed, we calculated the K_on,_ K_off_ and K_d_ of the evaluated molecules. The K_d_ values determined by the K_off_/K_on_ ratio show that the DFL20656 and the Merck compound 14 bind the receptor with a similar affinity, while the K_on_ values indicated that the orthosteric antagonist binds the B1 receptor more rapidly than the Merck and DFL20656 compounds but the most interesting observation is due to the K_off_ values indicating that DFL20656 and the orthosteric antagonist form more stable complexes than the Merck compound 14 with the receptor. Interestingly, molecules differed consistently in their receptor occupancy, with the Merck compound showing little persistence, 16 min, on the receptor compared to our molecule DLF20656, which rapidly bound the receptor and remained for 3 h (Table 1).

### 2.3. In Vitro Activity and Internalization

**Figure 5:**
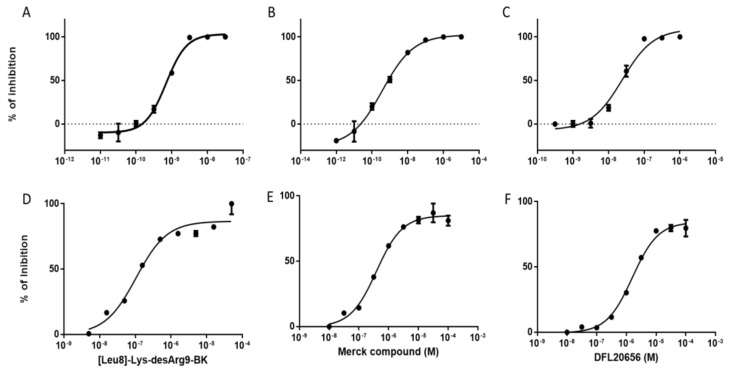
Selected compound activity in the intracellular calcium mobilization assay. Dose-response curves were generated to evaluate compound activity and to determine the IC_50_ both in the transfectant CHO-B1 (up) and in the IMR90 human cell line (down) physiologically expressing the B1 receptor. The orthosteric peptidic inhibitor [Leu^8^]-Lys-desArg^9^-BK (**A**,**D**), the Merck compound 14 (**B**,**E**) and Dompé DFL20656 (**C**,**F**) inhibited calcium release in CHO-B1 cells with pIC_50_ of 9.16 ± 0.05, 9.42 ± 0.07 and 7.75 ± 0.16 respectively. The inhibition of Ca^2+^ signaling was also observed in the IMR90 cell model with pIC_50_ values of 7.0, 6.4 and 5.8 respectively. The data represent the mean ± SEM of six experimental replicates.

The compound activity was evaluated in a dose-response curve by a calcium mobilization assay in Chinese hamster ovary cells (CHO) cells stably expressing the B1 receptor (Figure 5A–C) as well as on IMR90 human lung fibroblasts (Figure 5D–F) endogenously expressing the B1 receptor. We also tested the activity in rabbit aorta assay (Figure S3).

**Figure 6:**
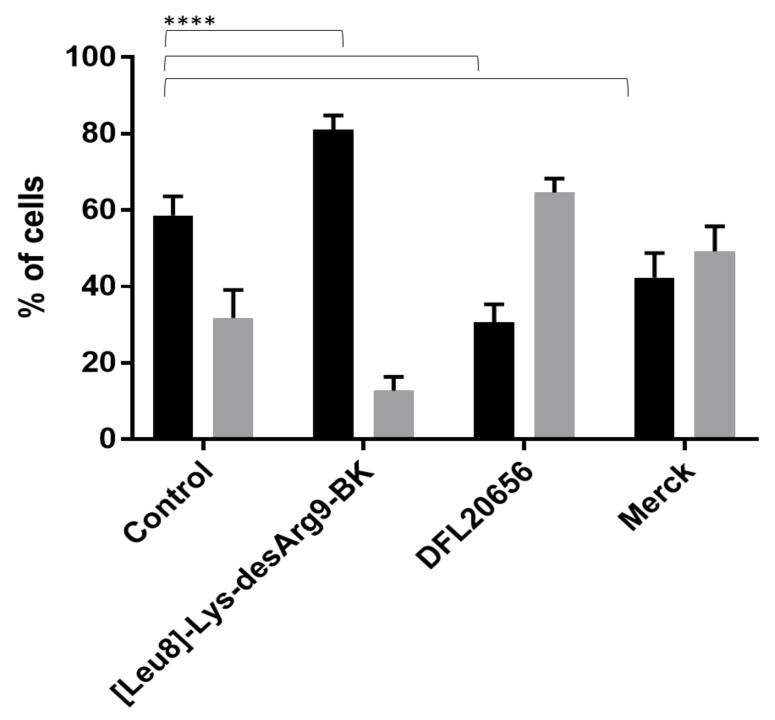
Analysis of B1 expressing cells: IMR90 cells were treated with selected inhibitors for 30′ at [2000 nM], subsequently the peptidic B1 agonist, Lys-desArg^9^-BK, was added and incubated for an additional 30′. Steady-state IMR90 (control) presented a distribution of B1 expressing cells (in black) and B1 negative cells (in grey). When antagonists were added the distribution changed. In particular, the peptidic antagonist [Leu^8^]-Lys-desArg^9^-BK, stabilized the presence of B1 upon the membrane while Dompé DFL20656 increased the percentage of B1-negative cells thus promoting B1 receptor internalization. The data represent the mean of 5 replicates. All data were presented as the mean ± SEM. Bonferroni-Dunn post hoc test was used. *p* < 0.05 was considered representative of significant differences. **** *p* < 0.0001.

The B1 receptor has been reported to be involved in the airway inflammation process and the IMR90 cell line represents a good physiological model to study the role of B1 [25]. The B1 receptor is weakly expressed by IMR90 under basal conditions, while it is strongly induced by treatment with the pro-inflammatory cytokine IL1β [25,26]. For our studies, we used IL1β-“activated” IMR90 cells. We evaluated the dose response of the DFL20656 compound (pIC_50_ = 7.8 and 5.8 in CHO and IRM90, respectively) in comparison with the peptidic antagonist of B1 receptor [Leu^8^]-Lys-desArg^9^-BK (pIC_50_ = 9.2 and 7.0 in CHO and IRM90, respectively), which is the reference standard for the analysis of B1 receptor activity and the Merck molecule (pIC_50_ = 9.4 and 6.4 in CHO and IRM90, respectively). To analyze the mechanism of action of selected molecules, we performed internalization studies in the IMR90 native cell model. IMR90 fibroblasts were pre-incubated for 30 min with selected antagonists and then treated with the peptidic agonist Lys-desArg^9^-BK for an additional 30 min. Then, the cells were stained and analyzed by flow cytometry. Interestingly, DLF20656 displayed internalization of the receptor in combination with the agonist Lys-desArg^9^-BK, while the Merck compound did not in an efficient way and the peptidic antagonist [Leu^8^]-Lys-desArg^9^-BK seemed to even stabilize the receptor in the membrane (Figure 6).

### 2.4. In Vivo Assays

An in vivo pharmacokinetic analysis of both DFL20656 and Merck compound 14 was carried out and the obtained pharmacokinetic parameters are presented in Table S1 (Supplementary Materials)

**Figure 7:**
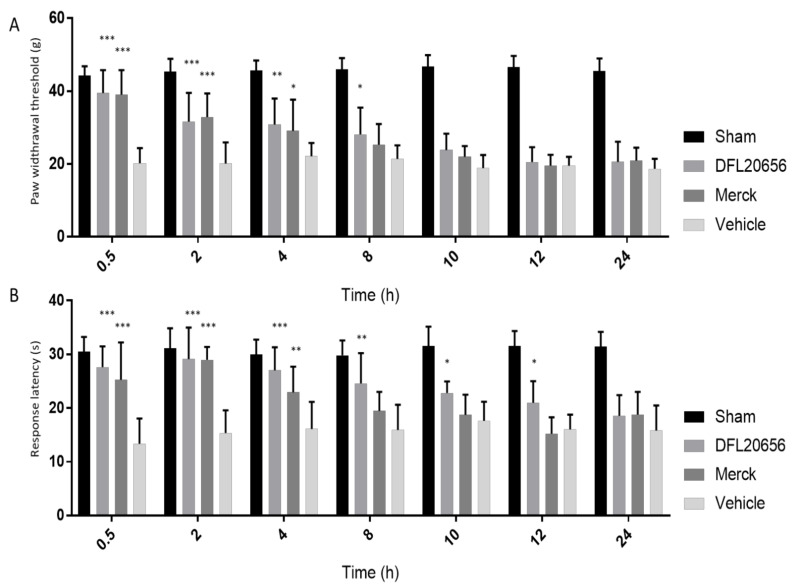
Antiallodynic effect of DFL20656 and Merck compound 14 after intravenous administration in a model of neuropathic pain. The efficacy of DFL20656 and the Merck compound were evaluated in a CCI rat model. Both mechanical allodynia (**A**) and thermal hyperalgesia (**B**) were evaluated. The DFL20656 showed a significant effect in reducing mechanical allodynia up to 8 h following intravenous administration, while Merck compound was active up to 4 h. For thermal hyperalgesia DFL20656 was active up to 12 h following administration, while Merck compound did not display significant efficacy after 4 h. The data represent the mean of 10 rats per experimental group. All data were presented as the mean ± SEM. Bonferroni-Dunn post hoc test was used. *p* < 0.05 was considered representative of significant differences. *** *p* < 0.001, ** *p* < 0.01 and * *p* < 0.05 vs CTR group.

In a chronic constriction injury (CCI) rat model, both mechanical allodynia and thermal hyperalgesia were evaluated following selected compound treatments. In mechanical allodynia experiments, the paw withdrawal latency of the animals treated with vehicle or our selected compounds was analyzed. On day 14 after sciatic nerve ligation, DFL20656 (10 mg/kg) reduced mechanical allodynia from 0.5 up to 8 h after iv administration, while the Merck compound (10 mg/kg) showed an antiallodynic effect from 0.5 up to 4 h after iv administration (Figure 7A). For thermal hyperalgesia, the response latency was observed in the Hargreaves apparatus. On day 14 after nerve injury, DFL20656 (10 mg/kg) significantly increased the response latency until 12 h, while the Merck compound elicited a significant response until 4 h (Figure 7B).

## 3. Discussion and Conclusions

Because of its role in chronic inflammation and pain processes, as well as in cancer progression, the B1 receptor has been in the last 30 years an attractive target for the pharma industry, which developed different peptidic and non-peptidic inhibitors of this receptor [13,27,28,29]. Several of these antagonists were investigated in clinical trials but the kallikrein-kinin system and its receptors are more complex than expected. Underestimated system complexity and few results translated from animal models to humans led to the failure of B1 inhibitors in the clinic. Only Icatibant (B2 antagonist) has been approved for the acute treatment of hereditary angioedema (HAE) [30]. An improved understanding of the mechanism of action of B1 inhibitors and their biological effects would be crucial for the development of antagonists able to successfully proceed in clinical trials. Here, we demonstrated the existence in the B1 receptor of a minor pocket that is able to bind small allosteric inhibitors, which, in addition to blocking Ca^2+^-mediated signaling, can modulate receptor activity through its internalization. For the first time, we elucidated the differences between the action of orthosteric peptidic B1 inhibitors and allosteric small molecules. Moreover, we unveiled differences between allosteric modulators’ biological activity with respect to their binding modes to the same allosteric site. We compared the orthosteric peptidic inhibitor [Leu^8^]-Lys-desArg^9^-BK with two allosteric small molecules that bind to the minor pocket identified in the B1 receptor in two different ways. Peptidic orthosteric inhibitor [Leu^8^]-Lys-desArg^9^-BK showed good receptor occupancy with a fast association with the B1 receptor and slow dissociation, resulting in a residence time of 2.5 h. The Dompé DFL20656 antagonist also manifested high receptor occupancy with an even longer residence time of approximately 3 h. Conversely, the Merck molecule showed a fast association but also a fast dissociation rate and a receptor occupancy of a few minutes (16 min). Single point mutagenesis in the minor pocket of the B1 receptor revealed that Dompé DFL20656 and Merck molecules bind to the same allosteric minor pocket on the receptor but involve different residues. The different binding modes resulted in a different biological effect; in fact, DFL20656 induced internalization in the presence of the agonist of B1, thus contrasting the effect of the peptidic agonist Lys-desArg^9^-BK, which binds the B1 receptor, stabilizing it on the cell membrane. For the first time, we analyzed this process in a native system such as IMR90 human lung fibroblasts, thus avoiding artefacts of transfected systems. The receptor internalization could be important for the prolonged efficacy displayed by DFL20656 in vivo in the CCI rat chronic pain model. In fact, in the rat model, DFL20656 was superior in efficacy with respect to the Merck compound 14, demonstrating significant efficacy in reducing mechanical allodynia until 8 h from compound administration, while its effect in reducing thermal hypersensitivity reached 12 h. The long-lasting efficacy in vivo is further supported by the long residence time evidenced in the radioligand binding experiment [31], where DFL20656 demonstrated a K_off_ of 0.0056 min^−1^. Altogether, these data suggested that an important feature of novel B1 receptor antagonists could be their ability to induce receptor internalization, in contrast to their stabilization in membrane induced by agonist binding under physio-pathological conditions. This characteristic, together with an extended residence time, could be required for B1 antagonists to be effective in human pathologies. Our pharmacological characterization of the different allosteric and orthosteric inhibitors highlighted the importance of having a wider characterization of active compounds in the early drug discovery processes because molecular activity in primary screening and their binding in the selected site are not sufficient to forecast their biological effects. The presented data revealed how different molecules bind and act differently on the B1 receptor in a native system and suggested that analysis of the mechanism of action is fundamental to developing new B1 receptor inhibitors for the clinic.

## 4. Materials and Methods

### 4.1. B1 Homology Model Generation and In Silico Molecular Docking Experiments

Due to the lack of a crystallographic model, a homology model of B1 receptor has been generated by using the GPCR-I-TASSER platform. Starting from the amino acid sequence downloaded from the Swiss-Prot database [32], GPCR-I-TASSER generated three-dimensional atomic models through multiple threading alignments and iterative structural assembly simulations [33]. After the generation of 5 theoretical models, the best one, selected according to specific parameters, was prepared by using Protein Preparation Wizard tool [34] integrated in Maestro Graphical User Interface (GUI) (Maestro, Schrödinger, LLC, New York, NY, USA, 2020). The target has been refined optimizing the hydrogen bonds and energy minimized by using OPLS_2005 [35] force field at physiological pH.

To obtain a reliable model, the B1 receptor was embedded into a palmitoyl phosphatidylcholine bilayer [36,37,38,39], by using the Orientations of Proteins in Membranes (OPM) server [40] to get the correct coordinates. After that, the Prime module has been used to optimize the loops and to minimize the structure, considering the membrane explicitly [41,42]. To overcome the bias of the templates used in the generation of the model, MD simulation was run allowing the homology model to equilibrate long enough to likely adopt what is more likely a biologically relevant structure [43]. To evaluate the structural stability of the protein-membrane system, 200 ns of molecular dynamics simulation were run [23,44].

MD calculation was carried out by using Desmond Multisim protocol [45]. The whole system was solvated in an orthorhombic box with a buffer of 10 Å TIP3 (transferable intermolecular potential 3-point) water molecules and were added counter ions to neutralize the system net charge. In the early stage, Multisim method allowed to equilibrate and relax the structures, simulating a mature system. The calculation was run under constant pressure of 1 atm and a temperature of 310 K, thermostated and barostated according to the Martyna-Tobias-Klein method, with a coupling constant of 0.5 (2.0) ps for the thermostat (barostat). The whole hydrogen positions were constrained by the M-SHAKE algorithm, allowing a time step of 2 fs. The long-range electrostatics were computed every time step by PME (Particle Mesh Ewald) method with a cut-off radius of 10Å. Docking calculations were performed using LiGen™ modules [46,47], an ensemble of tools for molecular de novo design, which can be used sequentially or as stand-alone program. These characteristics allow to easily define a customizable drug design workflow. In particular, LiGen™ Pass module, based on a new implementation of the algorithm used by PASS (putative active site with spheres) [48], was used to identify the protein binding site. Then, the LiGen™ Pocket module was used to analyze and characterize the binding site in terms of interaction points and the pharmacophore model was defined by donor, acceptor and hydrophobic points matching the key sites. Finally, a pharmacophore-based docking was performed by using the LiGen™ Dock module, which include a non-enumerative flexible docking algorithm. In the first step, the ligand pharmacophoric features were pre-computed then, the docked ligand is rotated in order to fit one pharmacophoric pocket point and the goodness of matching are evaluated. The ligand is subsequently turned to match a second pharmacophore feature, after that rotated by an appropriate angle (0.2 degrees) around the axis passing between the two-anchor points, trying to fit with a third feature. Thus, torsional angles are rotated of 0.5 degrees and ligand conformers are generated in situ trying to match as many features as possible. Each pose is scored by estimating the binding energy of the ligand-protein complex. Then, the theoretical affinity is compared with the scores of previously generated poses. If this actual score is better than the worst score of the already generated poses, the new pose is retained instead of the previous worst pose. More information about the docking procedure are reported in the work of Beato et al. [46].

### 4.2. Chemical Compounds Tested

DFL20656 is (R)-2-(2-(1H-imidazol-4-yl)-N-(2-methoxybenzyl)acetamido)-N-(((R)-tetrahydrofuran-2-yl)methyl)-2-(p-tolyl)acetamide and the Merck compound 14 is methyl (*R*)-4′-(1-((3-(2-cyanoacetamido)-4-methylpyridin-2-yl)amino)ethyl)-[1,1′-biphenyl]-2-carboxylate. Both compounds were synthesized at IRBM Pomezia (Rome), Italy.

[Leu^8^]-Lys-desArg^9^-BK is (2S)-2-[[(2S)-1-[(2S)-2-[[(2S)-2-[[2-[[(2S)-1-[(2S)-1-[(2S)-2-[[(2S)-2,6-diaminohexanoyl]amino]-5-(diaminomethylideneamino)pentanoyl]pyrrolidine-2-carbonyl]pyrrolidine-2-carbonyl]amino]acetyl]amino]-3-phenylpropanoyl]amino]-3-hydroxypropanoyl]pyrrolidine-2-carbonyl]amino]-4-methylpentanoic acid (CAS 71800-37-8), purchased from Sigma-Aldrich.

### 4.3. Radioligand Binding Assays

The effects of our negative allosteric modulators of the human B1 receptor on the binding of the natural orthosteric agonist desArg10-Kalleidin to the receptor (association kinetics, dissociation kinetics, equilibrium binding) were evaluated in two phases of radioligand binding experiments. The aim of phase 1 of the project was to evaluate the effect of the test compounds on the association kinetics of the radioligand and then to adjust the incubation time to equilibrium accordingly. The aim of phase 2 of the project was to evaluate the effect of the test compounds on the dissociation kinetics of the radioligand. The antagonist compounds’ names were unknown by the investigators who performed binding assays and data analysis.

Phase 1: Effect of allosteric test compounds on the association kinetics of the orthosteric radioligand [^3^H]desArg^10^-Kallidin. The B1 cell membranes were incubated with the orthosteric radioligand [^3^H]desArg^10^-Kallidin at 0.35 Nm in the absence or presence of B1 antagonists for 13 different incubation times: 5/10/15/20/30/45/60/90/120/180/240/360/480 min. Association curves were fitted and kobs values were calculated using Prism software.

Phase 2: Effect of allosteric compounds on the dissociation kinetics of the orthosteric radioligand [^3^H]desArg^10^-Kallidin. The B1 cell membranes were incubated with the orthosteric radioligand [^3^H]desArg^10^-Kallidin at 0.35 Nm in the absence or presence of the antagonists at 6 different concentrations: 0.01/0.1/1/10/100/1000 nM for 120 min to equilibrium binding as determined in the previous phase 1. The dissociation kinetics were then initiated by the addition of excess (10 μM) of unlabeled competitive orthosteric ligand, desArg9[Leu8]-Bradykinin and the incubations were then stopped after 13 different incubation times: 5/10/15/20/30/45/60/90/120/180/240/360/ 480 min. Dissociation curves were fitted and K_off_ values were calculated using Prism software. The kinetic binding data were calculated according to the equations reported in Supplementary Materials.

### 4.4. Cell Culture

Cellular biology experiments were performed in the native IMR90 human lung fibroblast system and in transfected CHO cells.

IMR90 cells were obtained from ATCCs and maintained in Minimum Essential Medium Eagle, EMEM (BioWhittaker, Lonza Group Ltd., Basel, Switzerland), supplemented with 10% foetal bovine serum (Euroclone, Pero, Milano, Italy), 100 units/mL penicillin/streptomycin, 2 mM UltraGlutamine (BioWhittaker). When required, IMR-90 cells were washed twice with 1X PBS (Gibco, Life Technologies, code 10010-023) and stimulated with 0.2 ng/mL human recombinant IL-1β (R&D SYSTEMS, code: 201-LB-025/CF) in serum-free EMEM (ATCC, code 30-2003) for 4 h at 37 °C.

CHO- B1 cells were grown in Dulbecco’s MEM/Nutrient Mix F12 (1:1) (BioWhittaker) supplemented with 1.35 mM sodium pyruvate, 11 mM hepes, 0.2% sodium bicarbonate, 10% foetal bovine serum (Euroclone), 100 units/mL penicillin /streptomycin, 2 mM UltraGlutamine (BioWhittaker), 1 mg/mL G418 sulfate and 5 µg/mL puromycin.

### 4.5. Wild-Type and Mutant B1 Constructs and Transfection

Human B1 (NM_000710) was amplified from human genomic DNA. A 1172 bp fragment of B1 cDNA containing the 1061 bp ORF was cloned and inserted into the pcDNA3 vector by using BamH1/XhoI restriction sites (pcDNA3_B1). B1 mutants were prepared using the pcDNA3_B1 construct as a template for site-directed mutagenesis to independently generate six different point mutations. All transient transfections were performed on 384-well plates using Lipofectamine 2000 (LifeTechnologies, Thermo Fisher Scientific Inc., Waltham, MA, USA) according to the manufacturer’s instructions. Briefly,10 μL Lipofectamine 2000 were diluted in 500 μL Opti-MEM^®^ (LifeTechnologies, Thermo Fisher Scientific Inc., Waltham, MA, USA) and incubated for 5 min at room temperature. In the meanwhile, 3 μg of plasmid DNA were diluted in 500 μL OPTIMEM (Invitrogen) and added to the Lipofectamine 2000 mix to obtain a final volume of 1 mL. After additional 20-30 min of incubation at room temperature, the 1 mL DNA-Lipofectamine 2000 complex was added to 1 mL of cell suspension (1.4 × 106 cells/mL) and cells were seeded at 17500 cells/well into Poly-D-Lysine coated 384-well plates (MATRIX black/clear bottom #4332-CPL, Thermo Scientific, Waltham, MA, USA). 4 h after transfection 25μL/well of medium containing 20% FBS were added.

All reactions were carried out using PfuUltra R High Fidelity DNA Polymerase AD (Stratagene, Agilent Technologies, Santa Clara, CA, USA) under conditions recommended by the manufacturer. All constructs were verified by sequencing.

### 4.6. Calcium Mobilization Assays

IMR90, CHO- BDKRB1 wild type and mutants were seeded 5000 cells/well in 384-well plates (MATRIX black/clear bottom #4332, Thermo Scientific, Waltham, MA, USA) in complete medium (25 µL/well). 24h after seeding, the cell plates were washed with Tyrode’s buffer by the Bio-Tek-ELx405 Microplate Washer (Merck Millipore; Billerica, MA, USA) and then loaded for 1h at rt by 10 µL/well of a solution containing the fluorescent Ca+2 indicator Fluo-4 NW dye. Increasing concentrations of antagonists were injected to the cells after basal Relative Fluorescence Units (RFU) signal setting (15000). Following ~30 min of incubation, a second injection of 25 µL/well of Lys-desArg^9^-BK reference agonist, at 3-fold concentration in Tyrode’s buffer/0.01% BSA (EC80: 70-100 nM final), was performed by the FLIPRTETRA. The signal of the emitted flash luminescence was recorded for 1 additional minute. The proper agonist EC80 was used for each construct based on the previously calculated EC50. Each compound was tested on all the bradykinin B1 receptor constructs and the empty pcDNA3 expression vector (MOCK) on the same plate, to properly compare the obtained results. Test compounds at 5-fold concentration respect to the final concentration to be tested (10 µM final, first injection) in 2.5% DMSO Tyrode’s buffer were added to the wells of the Assay Plates, in 10 µL volume (for a final DMSO concentration of 0.5%), by FLIPRTETRA (Molecular Devices, Sunnyvale, CA, USA). The kinetic response was monitored by the instrument over a period of 3.3 min. The bioactivity (response value in second injection, TA_KRV_Test-well_, in Relative Light Units (RLUs)) exerted by the compounds was expressed as percentage of inhibition. This value was computed relatively to the mean MAX controls response per plate corresponding to 0% inhibition (MAX_,_ (des-Arg^9^)-Bradykinin agonist at EC_80_, 70–100 nM) and the mean MIN controls signal response per plate (MIN_,_ Merck antagonist at IC_100_, 1 µM) corresponding to 100% inhibition, with the following formula:

$$PercentInhibition\;=\;100\;\times \;\left(1-\frac{TA\_KRV_{Test\_well}-\bar{MIN}}{\bar{MAX}-\bar{MIN}\;}\right)$$

The overall Z’ factor mean for the screen, which reflects the plate quality criteria, was >0.8, a value suitable for HTS. Hits were classified as test compounds that inhibited the EC80 agonist signal by >22%, as compared with a no-inhibitor control and were confirmed after test at increasing concentrations, in order to determine IC_50_.

### 4.7. Flow Cytometry of IMR-90 Cells

IMR-90 cells were cultured according the specifics reported above. The day of the experiment, IMR-90 cells were washed twice with 1X PBS (Gibco, Life Technologies, code 10010-023) and stimulated with 0.2 ng/mL human recombinant IL-1β (R&D SYSTEMS, code: 201-LB-025/CF) in serum-free EMEM (ATCC, code 30-2003) for 4 h at 37 °C. After stimulation, cells were co-incubated with the EC80 B1 agonist Lys-desArg^9^-BK (30 nM) and antagonists at 2000 nM in serum-free EMEM for 30′. After agonist/antagonist stimulation, IMR90 cells were fixed in ice-cooled methanol at −20 °C for 3 min and then harvested by scraping. Subsequently, the cells were stained as follows. The primary antibody used for BDKRB1 staining was a bradykinin B_1_R antibody (N-18) (Santa Cruz Biotechnology, code: sc-15041, Dallas, TX, USA). The primary antibody was diluted 1:50 in 1X PBS and incubated overnight at 4 °C. Then, IMR-90 cells were washed with 1X PBS and incubated for 45 min with an Alexa Fluor^®^ 647 conjugate secondary antibody (Invitrogen, code: A-21447, diluted 1:500). Dead or damaged cells were stained with LIVE/DEAD™ Fixable Aqua Dead Cell Stain Kit for 405 nm excitation from Invitrogen (Invitrogen cat No. L34965) according to the manufacturer’s instructions. Data were acquired using a BD Influx (BD Biosciences, San Jose, CA, USA) and analysed with FACS Diva software. Investigators who performed data analysis were not aware of the identity of the antagonists’ compounds, both allosteric and orthosteric.

### 4.8. In Vivo Compound Activity Evaluation

#### 4.8.1. Animals

The behavioral experiments were performed on male Sprague–Dawley rats (7–9 weeks of age; Charles River, Italy) housed in the animal care facility of the Department of Pharmacy of the University of Naples, Italy. Animals were housed in groups of five, in a room with controlled temperature (22 ± 1 °C), humidity (60 ± 10%) and light (12 h per day); food and water were available ad libitum throughout the study. All animals were weighed on the day of each treatment. All behavioral tests were performed between 09:00 and 17:00 h and the animals were used only once. Animal care and manipulations were conducted in conformity with International and National law and policies (EU Directive 2010/63/EU for animal experiments, 22th September 2010, ARRIVE guidelines and the Basel declaration including the 3R concept). The procedure reported here was approved by the Institutional Committee on the Ethics of Animal Experiments (CVS) of the University of Naples Federico II and by Ministero della Salute under protocol no. 2014-00884607. Animal studies are reported in compliance with the ARRIVE guidelines (Kilkenny et al., 2010; McGrath and Lilley, 2015). Each animal was uniquely identified with a colored spray on the back before the experiment.

#### 4.8.2. Experimental Groups and Procedures

For the behavioral experiments, rats were randomized and divided into equal-sized groups (*n* = 10 per group) not predetermined by a statistical method. Animals were treated with vehicle, DFL20656 (10 mg·kg^−1^, i.v.) and Merck compound (10 mg·kg^−1^, i.v.). The volume of liquid administered was 0.3 mL. Details of the anesthesia are provided below. At the end of the procedures, the animals were killed by cervical dislocation.

#### 4.8.3. Drug Treatment

DFL20656, 10 mg/kg/iv and Merck compound, 10 mg/kg/iv were dissolved in PEG400/water (50/50, *v*/*v*). PEG400 (50% of volume) was added to the compound, after allowing to stir for 15 min at room temperature, was added water (the other 50% of volume) and after 15 min under stirring a clear solution was obtained.

#### 4.8.4. Chronic Constriction Injury (CCI) Model of Neuropathic Pain

Neuropathic pain behavior was induced by ligation of the sciatic nerve according to the method described by Bennett and Xie [49]. Briefly, rats were anaesthetized (100 mg/kg ketamine and 10 mg/kg xylazine i.p.) and the left sciatic nerve was exposed at the level of the thigh by blunt dissection through the biceps femoris. Proximal to the sciatic’s trifurcation, about 12 mm of nerve was freed of adhering tissue and four ligatures were loosely tied around it with about 1 mm spacing so that the epineural circulation was preserved. Sham animals represent rats operated but not ligated.

#### 4.8.5. Mechanical Allodynia (Von Frey Test)

To assess for changes in sensation or in the development of mechanical allodynia, sensitivity to tactile stimulation was measured using the Dynamic Plantar Aesthesiometer (DPA, Ugo Basile, Italy). Ligated animals were placed in a chamber with a mesh metal floor covered by a plastic dome that enabled the animal to walk freely but not to jump. The mechanical stimulus (paw withdrawal threshold) was then delivered in the mid-plantar skin of the hind paw. The cut-off was fixed at 50 g, while the increasing force rate (ramp duration) was settled at 20 s. The DPA automatically records the force at which the foot is withdrawn and the withdrawal latency. Each paw was tested twice per session. This test did not require any special pre-training, just an acclimation period to the environment and testing procedure. Test was performed form 0.5 h up to 24 h after iv administration, on the ligated paw at days 7 and 14 after ligation.

#### 4.8.6. Thermal Hyperalgesia (Plantar Test, Hargreaves Apparatus)

Heat hypersensitivity was assessed using the rat plantar test apparatus following method described by Hargreaves et al. [50]. The plantar test consisted of three Perspex boxes (22 × 19 × 25 cm) on an elevated glass table. Rats were housed in each box, so that 3 rats could be tested simultaneously in a single apparatus and left to acclimatize for at least 10 min. A mobile infrared heat source was applied to the plantar surface of the hind paws. The paw withdrawal latency was defined as the time (expressed in seconds) taken by the rat to remove its hind paw from the heat source. The heat source was calibrated to 15 IR intensity and cut off point of 50 s was applied to prevent tissue damage. Test was performed form 0.5 h up to 24 h after iv administration, on the ligated paw at days 7 and 14 after ligation.

### 4.9. Statistical Analysis

All the experimental data were analyzed by a computer fitting procedure (GraphPad Prism 6.0, La Jolla, CA, USA, RRID:SCR_002798) and expressed as the mean ± SEM or fold change of at least five independent experiments. All the group sizes were designed to be homogeneous. ANOVA and Student’s *t*-test were selected for statistical analyses. When required, the Bonferroni-Dunn post hoc test was used. *p* < 0.05 was considered representative of significant differences.

## Figures and Tables

**Table 1:** Summary of kinetic binding data for the compounds analyzed. For Merck compound 14, DFL20656 and [Leu^8^]-Lys-desArg ^9^-BK, *n* = 5 independent experiments.

Compound	K_off_(min^−1^)	Residence Time (min)	K_on_ (M − 1 min^−1^)	K_i_[K_d_](M)
Merck compound 14	6.08 × 10^−2^ ± 9.78 × 10^−3^	19.06 ± 3.41	3.92 × 10^7^± 3.54 × 10^6^	1.53 × 10^−9^ ± 1.89 × 10^−10^
DFL20656	5.34 × 10^−3^ ± 5.66 × 10^−4^	198.14 ± 20.41	5.06 × 10^6^ ± 2.57 × 10^5^	1.05 × 10^−9^ ± 8.44 × 10^−11^
[Leu^8^]-Lys-desArg^9^-BK	5.83 × 10^−3^± 3.93 × 10^−4^	176.04 ± 13.08	4.87 × 10^7^± 1.92 × 10^6^	1.21 × 10^−10^ ± 9.57 × 10^−12^

K_off_: Constant of Dissociation; K_on_: Constant of Association; K_i_: Constant of Inhibition; K_d_: Constant of Displacement.

## Supplementary Materials

Supplementary materials can be found at https://www.mdpi.com/1422-0067/21/20/7677/s1.

## Abbreviations

TM	Transmembrane
GPCRs	G-protein-coupled receptors
CXCR1	C-X-C Motif Chemokine Receptor 1
CXCR2	C-X-C Motif Chemokine Receptor 2
C5a	Complement component 5a
I-TASSER	Iterative Threading ASSEmbly Refinement
MD	Molecular Dynamics
RMSD	Root mean square deviation
CHO	Chinese hamster ovary cells
CCI	Chronic constriction injury
HAE	Hereditary angioedema
OPM	Orientations of Proteins in Membranes
PME	Particle Mesh Ewald
RFU	Relative Fluorescence Units
PASS	Putative active site with spheres
