# Supplementary material for: Binding Mode Exploration of B1 Receptor Antagonists’ by the Use of Molecular Dynamics and Docking Simulation—How Different Target Engagement Can Determine Different Biological Effects
Source: Int J Mol Sci. 2020 Oct 16;21(20):7677. doi: 10.3390/ijms21207677 (PMC7590058; doi:10.3390/ijms21207677)
Supplement: Supplementary file 1 [file ijms-21-07677-s001.pdf]

Supplementary Material

Binding Mode Exploration of b1 Receptor Antagonists′ by the Use of Molecular Dynamics and Docking Simulation—How Different Target Engagement Can Determine Different Biological Effects

Pharmacokinetic Analysis

The pharmacokinetics of DFL20656 and the Merck compound were investigated after intravenous administration (Table S1). Following intravenous administration of 10 mg/kg in rats, the DFL20656 concentration over 8 h ranged from 35.7 to 0.4 μg/mL with a Cmax of 35.7 μg/mL. The Merck compound showed a concentration in plasma ranging from 32.6 to 0.01 μg/mL with a Cmax of 32.6 μg/mL. The two products also showed similar elimination kinetics with similar Cmax and T1/2 (0.73 and 0.79 for DFL20656 and Merck compound 14, respectively). The two compounds showed a low clearance and a high apparent distribution volume at the steady state. DFL20156 showed a slightly longer mean residence time of 1.15 h with respect to 0.86 h of Merck compound 14 (Table S1).

Table 1. Pharmacokinetic parameters.

Parameter.	Unit	Mean	SD
**Merck compound 14**			
**t1/2**	hr	0.79	0.04
**Tmax**	hr	0.08	0
**Cmax**	μg/ml	32.61	14.49
**C0**	μg/ml	41.24	22.82
**Tlast**	hr	8	0
**Clast_obs**	μg/ml	0.01	0.010
**AUC 0-t**	μg/mL *hr	24.99	13.72
**AUC 0-inf**	μg/mL *hr	25.01	13.74
**MRT 0-t**	hr	0.86	0.38
**MRT 0-inf**	hr	0.86	0.38
**Cl_obs**	mL/Kg *hr	519.7	342.2
**Vss_obs**	L/kg	374	97
**DFL 20656**			
**t1/2**	h	0.73	0.08
**Tmax**	h	0.08	0.00
**Cmax**	μg/mL	35.70	15.74
**C0**	μg/mL	38.77	18.81
**Tlast**	h	8	-
**Clast_obs**	μg/mL	0.04	0.03
**AUC 0-t**	μg/mL*h	53.90	30.78
**AUC 0-inf**	μg/mL*h	53.95	30.81
**MRT 0-t**	h	1.15	0.08
**MRT 0-inf**	h	1.15	0.07
**Cl_obs**	mL/kg *h	274	233.8
**Vss_obs**	mL/kg	311	254

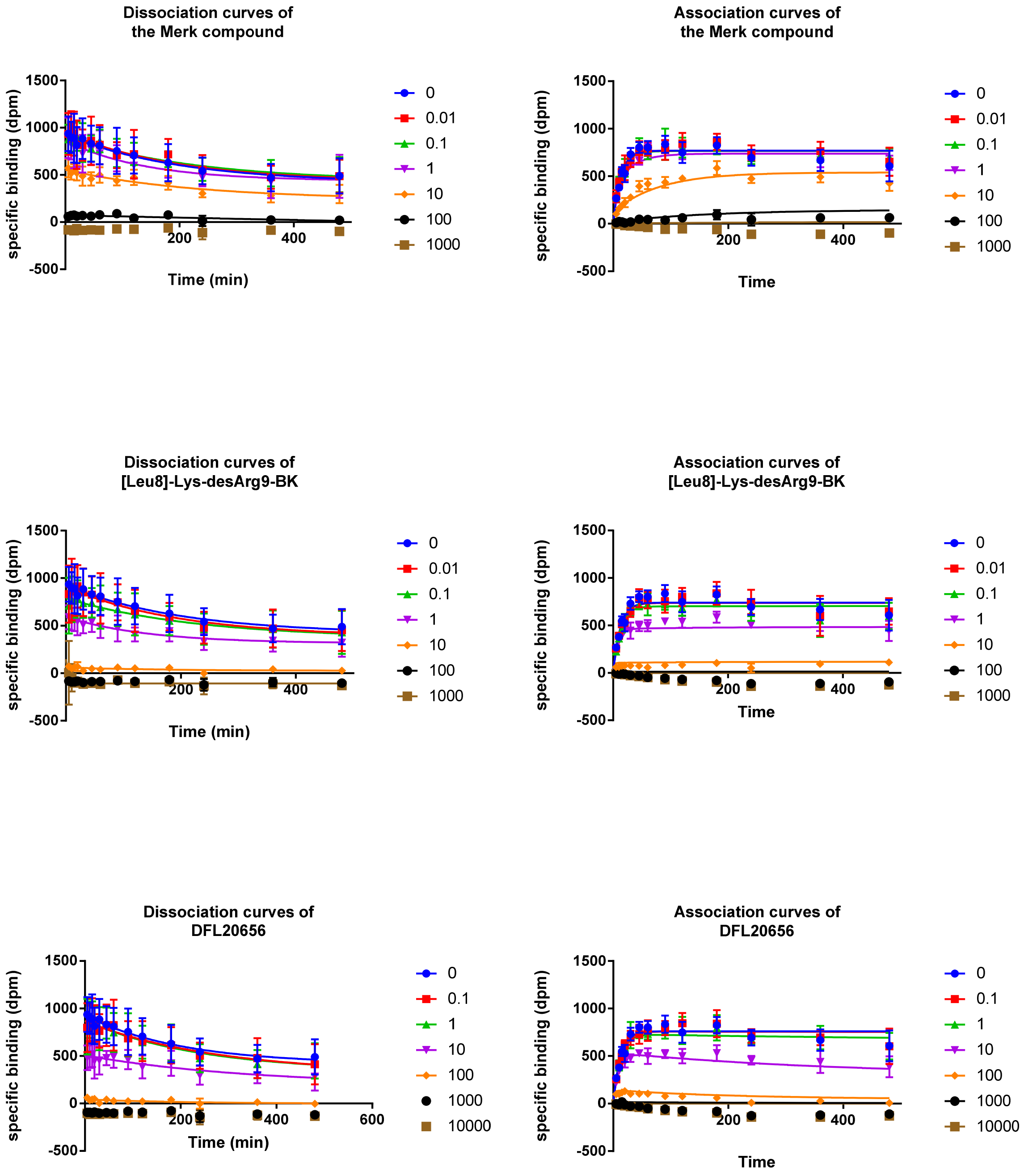

Figure 1. Association and dissociation curves of the Merck compound, DFL20656 and [Leu[8]]-Lys-desArg[9]-BK: for each compound the association and dissociation curves calculated at seven concentration points and thirteen timepoints are reported. The figure shows the mean and SEM of 5 independent experiments in duplicate.

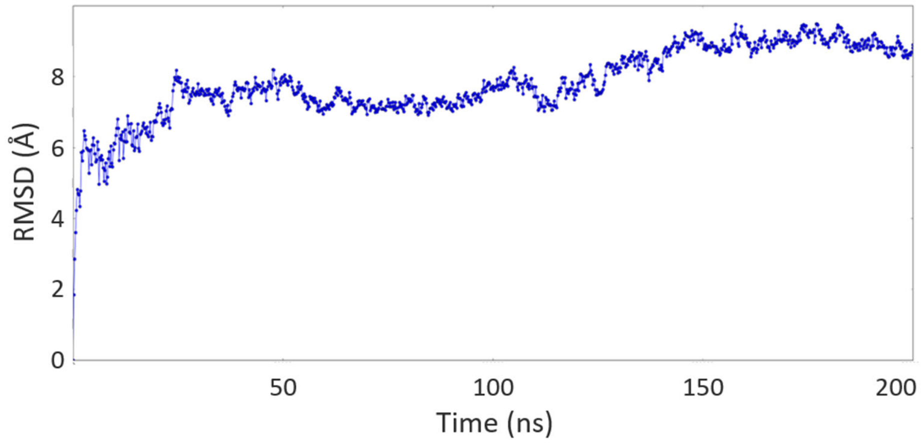

Figure 2. The RMSD trend calculated on the backbone atoms, during 200 ns of MDs.

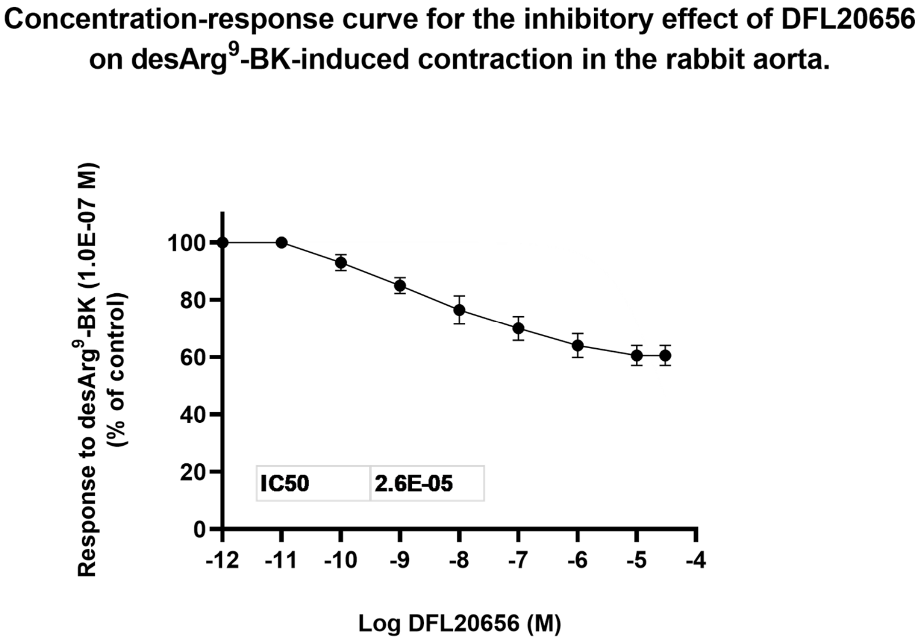

Figure 3. DFL20656 activity in rabbit aorta assay. In the rabbit thoracic aorta, DFL20656 at a concentration as high as 3.0E-05 M, did not induce a contraction but inhibited the response desArg9-BK. The following results indicate that DFL20656 behaves as an antagonist at the Bl receptors in this tissue. Data shown are the mean of a triplicate experiment.

Supplemental Methods

Radioligand Binding Assays

The kinetic binding data were calculated as follows:

Calculation of Kobs and association half-life values using prism software

$Y = Ymax \times (1 - \exp(-1 * Kobs * X))$

Kobs is the observed association rate constant in units of M−1 min−1

Ymax is the maximum plateau at equilibrium in the unit of the y axis.

The half-life equals ln(2) divided by Kobs.
Calculation of koff (= k2) values using prism software
Y= (Y0 − NS)*exp(− K*X) + NS
Y0 is the binding at time zero in the units of the Y axis.
NS is the non-specific binding at infinite times in the units of the Y axis.
Koff is the rate constant in inverse units of the X axis.
The half-life equals ln(2) divided by Koff.
Calculation of Kon values with Excel software
The association rate constant, Kon, is calculated using the following equation, where the Koff value used is predetermined from dissociation rate experiments:
Kon (M-1.min − 1) = Kobs (min − 1) − Koff (min − 1)/ [L] * (M)[L] = radioligand concentration
Kd (M)= Koff (min − 1)/ Kon (M − 1.min − 1)
Calculation of Kon (= k3) and Koff (= k4) values of unlabeled antagonist compounds using prism software
The Kon and Koff values of the radioligand are determined directly from the association and dissociation curves, respectively, using prism software. Subsequently, the Kon and Koff of cold antagonists were determined as follows:
Ka = K1*[L]*1 − 9+ K2
Kb = K3*[I]*1 − 9 + K4
S = SQRT[(Ka − Kb)2 + 4*k1*k3*L*I*1 − 18]
Kf = 0.5*(Ka + Kb + S)
Ks = 0.5*(Ka + Kb − S)
Q = Bmax*K1*L*1 − 9/Kf − Ks
Y = Q*[K4*(Kf − Ks)/ /(Kf*Ks)+[(K4 − Kf)/Kf]*exp(− Kf*X) − [(K4*Ks)/Ks]*exp(− Ks*X)]

Pharmacokinetics

Sprague Dawley male rats (body weights 250 g at the time of the treatment) were used in this study. The animals were originally supplied by Harlan, Italy. The animals were housed, in a group of four, in cages suitable for the species, also during dosing and feeding periods. The animals were housed in a single, exclusive room, air conditioned to provide a minimum of 15 air changes/hour. The environmental controls were set to maintain temperature within the range 22°C and relative humidity within the range 50 to 60% with an approximate 12 h light and 12 h dark cycle that is controlled automatically. Food and water were available ad libitum throughout the study. All animals were weighed on the day of the treatment. Clinical signs were monitored at regular intervals throughout the study in order to assess any reaction to treatment. The experiment was carried on in agreement with the Italian Law D. L.vo 4 marzo 2014, n. 26 Blood samples were collected in heparinized eppendorfs (Heparin Vister 5000 U.I/mL), gently mixed and placed immediately on ice; then eppendorfs were centrifuged (3500 × g, at 4 °C for 15 min) and the resulted plasma collected and transferred to uniquely labelled eppendorfs and frozen at – 20 °C till the analysis. At the end of the study animals were sacrificed by exsanguination under deep isoflurane anesthesia. After the bolus, blood (approximately 100ul) was sampled from tail vein at the following timepoints: 5, 15, 30, 60, 120 240, 480 min and 24 h. Samples were analyzed on UPLC (Acquity, Waters) coupled with a API 3200 Triple Quadrupole AB Sciex. Non compartmental analysis was applied and the following PK parameters were evaluated for each subject—Maximum plasma concentration (Cmax), Plasma concentration at last timepoint (Clast), Time of maximum plasma concentration (tmax), AUC from time zero to the time of the last quantifiable plasma concentration (AUC0-last), AUC from time zero extrapolated to infinity (AUCinf), Mean Residence Time (MRT), Half-life (T½), Clearance Dose/AUC (Cli), Apparent distribution volume at the steady-state (Vss): Dose*AUMCinf/(AUCinf)2. Concentration data were extrapolated using the software Analyst™ 6.1 (Applied Biosystems); AUCs were calculated by linear trapezoidal rule and a uniform weight was performed as a first general approach. Graphical concentration-time curves are produced after Log transformation. Softwares (PK Solver 2.0, Excel 2007 Microsoft add in).

Rabbit Thoracic Aorta Assay

Rings of rabbit thoracic aorta denuded of endothelium were suspended in 20 mL organ baths filled with an oxygenated (95% O_2 and 5% CO_2) and pre-warmed (37 °C) physiological salt solution of the following composition (in nM): NaCl 118.0, KCl 4.7, $MgSO_4$ 1.2, $CaCl_2$ 2.5, KH_2PO_4 1.2, $NaHCO_3$ 25 and glucose 11.0 (pH 7.4). The tissues were

connected to force transducers for isometric tension recordings. They were stretched to a resting tension of 4 g, washed several times then allowed to equilibrate overnight. The experiments were carried out using semi-automated isolated organ systems possessing eight organ baths, with multichannel data acquisition. The tissues are exposed to a submaximal concentration of the reference agonist Lys-desArg9-BK (0.1 μM) to obtain a control contractile response. After stabilization of the agonist-induced response, the tissues are exposed to increasing concentrations of the antagonists. The concentrations are added cumulatively and each is left in contact with the tissues until a stable response is obtained or for a maximum of 30 min. The parameter measured is the maximum change in tension induced by each compound concentration. The results are expressed as a percent of the control response to Lys-desArg9-BK.
